# The Association of Obesity with Autoimmune Thyroiditis and Thyroid Function-Possible Mechanisms of Bilateral Interaction

**DOI:** 10.1155/2020/8894792

**Published:** 2020-12-14

**Authors:** Agnieszka Baranowska-Bik, Wojciech Bik

**Affiliations:** ^1^Department of Endocrinology, Centre of Postgraduate Medical Education, Ceglowska 80, Warsaw 01-809, Poland; ^2^Department of Neuroendocrinology, Centre of Postgraduate Medical Education, Marymoncka 99/103, Warsaw 01-813, Poland

## Abstract

A growing number of patients suffer from autoimmune diseases, including autoimmune thyroid disease. There has simultaneously been a significant increase in the prevalence of obesity worldwide. It is still an open question whether adiposity can directly influence activation of inflammatory processes affecting the thyroid in genetically predisposed individuals. Adipokines, biologically active substances derived from the adipocytes, belong to a heterogenic group of compounds involved in numerous physiological functions, including the maintenance of metabolism, hormonal balance, and immune response. Notably, the presence of obesity worsens the course of selected autoimmune diseases and impairs response to treatment. Moreover, the excess of body fat may result in the progression of autoimmune diseases. Nutritional status, body weight, and energy expenditure may influence thyroid hormone secretion. Interestingly, thyroid hormones might influence the activity of adipose tissue as metabolic alterations related to fat tissue are observed under pathological conditions in which there are deficits or overproduction of thyroid hormones. Functioning TSH receptors are expressed on adipocytes. Thermogenesis may presumably be stimulated by TSH binding to its receptor on brown adipocytes. There could be a bilateral interaction between the thyroid and adipose. Obesity may influence the onset and course of autoimmune disease.

## 1. Introduction

An abundance of data has indicated a growing obesity epidemic in the last few decades. A wide range of various comorbidities known as associated to obesity includes metabolic abnormalities (insulin resistance, type 2 diabetes mellitus, and nonalcoholic fatty liver disease), immune-mediated diseases, and some types of cancer [1]. At the same time, a rise in the prevalence of autoimmune disorders, including thyroid autoimmunity, has been observed [2].

Adiposity, especially when the fat depots are located in the visceral region of the body, is correlated with chronic low-grade inflammation [3]. Moreover, in the case of obesity, the secretion of adipocyte-derived molecules called adipokines, as well as of cytokines, is disturbed [4]. The improper release of adipokines and cytokines consequently leads to impaired metabolism and enhances the inflammatory state [4]. Obesity also results in morphological changes in adipose tissue itself, including adipocyte hypertrophy, altered phenotype, and localization of immune cells, as well as vascular and structural cells [3, 4]. Noticeably, the activity of adipose tissue varies depending on the fat depot's location with visceral adipose tissue being the most biologically active [4].

Interestingly, in the past, evolutionary processes favored individuals who were able to perform gluconeogenesis and develop insulin resistance, promoting a thrifty genotype with the accumulation of fat deposits. Moreover, obesity with related inflammatory responses and innate immunity helped to counteract infection and allowed for survival despite famine and infections [5].

Two best-known adipokines are leptin and adiponectin. The secretion of leptin positively correlates with body mass index that serves as an indicator of body fat amount [6]. There are differences in leptin concentrations depending on gender with much higher levels found in females than in males [7].

Clinical and experimental data presented in the literature strongly support the involvement of adipokines in the pathogenesis of immune-mediated disorders [4]. It has been suggested that obesity could be related to a higher risk of rheumatoid arthritis, psoriasis and psoriatic arthritis, systemic lupus erythematosus, multiple sclerosis, and possibly thyroid immunity including Hashimoto's thyroiditis [1, 8]. Additionally, obesity has been associated with more severe forms of autoimmune diseases with reduced therapeutic response to typical treatment [1].

Autoimmune thyroid diseases (AITDs) belong to the group of diseases, resulting from the changes in immune tolerance and autoimmune-based degradation of tissues. AITDs comprise different types of thyroiditis, predominantly Hashimoto's thyroiditis (HT) and Graves' disease. Hashimoto's thyroiditis is believed to be a widespread autoimmune disease and the most common cause of hypothyroidism [9–13]. In this review, the focus will be on Hashimoto's thyroiditis as the autoimmune thyroid disease of interest. The incidence of autoimmune thyroid diseases varies depending on the geographic region, iodine uptake, sex, and other factors. Hashimoto's thyroiditis is 8 times more prevalent in women than in men [8, 11].

Hashimoto's thyroiditis was described for the first time in 1912 by Dr. Hakaru Hashimoto and initially was named lymphadenoid goiter [14]. After several years of studies, the autoimmune etiology of this kind of thyroiditis was elucidated [14]. Despite the intensive evaluation, the exact etiopathogenesis of HT has not been fully understood to this day [8]. However, it is widely accepted that autoimmune thyroid disease has a multifactorial origin with the role of endogenous as well as exogenous factors [15, 16]. A complex interaction between genetic and environmental factors may trigger HT. Notably, hypotheses are suggesting a relationship between thyroid diseases and/or thyroid function and obesity [17]. As mentioned before, a significant increase in the prevalence of both, autoimmune thyroiditis and obesity, is noticed worldwide [18, 19]. However, it is still an open question of whether an excess of fat tissue directly affects the activation of the inflammatory process in the thyroid of genetically predisposed individuals [17].

On the other hand, thyroid hormones might influence the activity of adipose tissue. Thus, it could be supposed that there is a bilateral interaction between thyroid and adiposity.

Therefore, we present a wide spectrum of knowledge from the theoretical and clinical points of view concerning a potential relationship between thyroid autoimmunology/thyroid function and adiposity. The decision for exploring these two issues, thyroid autoimmunity-obesity and thyroid function-obesity correlations, at the same time, was based on the findings that obesity is related to chronic low-grade inflammation. Therefore, obesity-induced pathological processes might initiate the autoimmune cascade and, consequently, may impact both autoimmunological response and thyroid hormone production. In our opinion, this paper is unique in its scope. This review aims to summarize the existing data extensively. Such a broad approach to given topics will allow the readers to understand the mechanisms of potential processes occurring between the thyroid gland and adipose tissue. Moreover, we believe that this paper could indicate a novel research possibility in experimental medicine. Finally, the current knowledge presented in this review may be relevant in clinical practice in terms of appropriate communication between the clinician and patient. Nowadays, patients are looking for an explanation for their obesity or metabolic disorders, and they often believe that obesity is related to the thyroid disease itself. Therefore, clinical practitioners should know whether there are interactions between thyroid and adipose tissue to explain the doubts. This knowledge might also help to make the right therapeutic decision, e.g., not to treat obesity with l-thyroxine.

## 2. The Role of Adipose Tissue in Autoimmune Processes

An increased amount of adipose tissue leads to not only structural changes of adipocytes and altered adipokines secretion but also has immunological consequences [4]. Firstly, in the course of obesity, increased adipokine and cytokine release from white adipose tissue, especially these molecules with modified profile towards proinflammatory activity, promotes the recruitment of additional cytokine-producing immune cells and intensifies existing low-grade systemic inflammation [20]. It is widely accepted that white adipose tissue is a significant source of cytokines and chemokines, including, among others, interleukin-6 (IL-6) and tumor necrosis factor-alpha (TNF-*α*), the well-known proinflammatory regulators [21]. Subsequently, an association between adiposity and its participation in the progression of autoimmune inflammatory diseases has been discussed in the literature [8]. Moreover, obesity is correlated with many immune cells associated with a proinflammatory phenotype, including Th17 cells [20, 22]. Besides, not only obesity itself but also high fat intake promotes inflammation by affecting the signaling of toll-like receptors (TLRs) [23]. According to the current knowledge, TLRs are mediators of innate immunity and they also play an important role in adaptive immunity by promoting proinflammatory cytokines and upregulating stimulation of antigen-presenting cells [23, 24]. Furthermore, an enhanced infiltration of adipose tissue by T lymphocytes and macrophages is observed in obesity, and these cells are regulated by TLRs, whose dysregulation in signaling, in turn, triggers autoimmunity [23].

Noticeably, as mentioned before, adipokines are active players in inflammatory disorders. Although the adipokine family comprises a large number of different bioactive molecules [25], we will only focus on leptin, one of the best-known factors participating in inflammatory processes and autoimmunity.

Leptin is an adipokine that links metabolism and immune homeostasis as it possesses the ability to influence energy balance and has an impact on the immune system [26]. Leptin upregulates secretion of IL-6, IL-12, and TNF-*α*, particularly from white adipose tissue [27]. The role of leptin in *B*-cell homeostasis via suppression of apoptosis, induction of cell cycle, and mediation of the release of pro- (TNF-*α* and IL-6) and anti-inflammatory (IL-10) cytokines has also been reported [4, 28]. Interestingly, leptin could also influence communication between the cells of the immune system, including *B* and *T* lymphocytes that perform the direct actions in adaptive immune responses [26].

Leptin exerts both direct and indirect proinflammatory effects in the body and shows pluripotent activity in the field of the innate and adaptive immune mechanisms.

When considering innate immunity, the presence of leptin launches several mechanisms influencing the production of proinflammatory molecules and immune signaling cascades.

Leptin plays a role in neutrophil recruitment. It mediates neutrophil chemotaxis and infiltration. Besides, leptin induces the survival of neutrophils. Of note, individuals suffering from obesity and, simultaneously, hyperleptinemia demonstrated changed properties of neutrophils with enhanced superoxide release and chemotactic activity [4, 29–31].

Leptin affects the expression of adhesion molecules on eosinophils as well as the secretion of proinflammatory factors from eosinophils. In detail, leptin enhances the release of proinflammatory mediators (IL-1*β*, IL-6, IL-8, and MCP-1 (monocyte chemoattractant protein-1)) and upregulates the expression of cell surface adhesion molecules ICAM-1 and CD18 while downregulating ICAM-3 and *L*-selectin in eosinophils [4, 32]. Additionally, leptin affects also eosinophil survival by delaying apoptosis. In children and adolescents with enormous adiposity (defined according to the National Centre for Health Statistics, as a body mass index above 95 percentile of BMI curve) and hyperleptinemia, eosinophils demonstrated greater adhesion and chemotaxis than normally [33].

Furthermore, it has been revealed that in basophils the leptin-induced migratory activity promotes IL-4 and IL-13 secretion, increases the cell surface expression of CD63, and augments cell degranulation in response to the aggregation of IgE [4, 34].

Interestingly, leptin has also an impact on macrophage activation and phagocytosis [4]. According to the experimental studies on macrophages, leptin treatment promotes phagocytic activity via modulation of cAMP levels, intracellular ROS (reactive oxygen species) generation, and chemotactic responses through intracellular calcium influx, JAK/STAT (Janus kinase/signal transducers and activators of transcription), MAPK (mitogen-activated protein kinase), and PI3K (phosphoinositide 3-kinase) signaling pathways [35–37]. Although human adipose tissue macrophages (ATM) exposed to leptin in culture expressed surface markers that were more similar to the M2 phenotype (anti-inflammatory, alternatively activated), they were also able to produce TNF-*α*, IL-6, IL-1*β*, IL-1ra, IL-10, MCP-1, and MIP-1*α* (macrophage inflammatory proteins-1*α*) typical for M1 cells (classically activated) that encourage inflammation [38]. These results of mixed M1/M2 spectrum could be explained by the fact that macrophages in obese adipose tissue display surface proteins that resemble neither classical nor alternative activation, but rather represent a state of metabolic activation [39].

Moreover, it has been speculated that leptin could initiate the recruitment of macrophages to adipose tissue [40].

Additionally, leptin influences NK (natural killer) cells modulating its activation. Detailed leptin action involves enhancing immature NK survival through Bcl-2 and Bax gene modulation and increasing NK cytotoxicity via STAT3 activation and expression of IL-2 and perforin [41, 42]. Interestingly, long-term exposure to leptin, similar to the situation in obese individuals in whom elevated serum leptin levels are seen, significantly impairs integral parts of NK cell immune functions and decreases cell proliferation while short-time stimulation with leptin increases IFN-*γ* (interferon *γ*) secretion, CD69 activation marker expression, and cytotoxic lysis of tumor cells [43]. Besides, NK function in obese individuals is impaired when compared to lean subjects, probably as an effect of leptin resistance [44].

Leptin has also been found to act as an activator of human dendritic cells (DCs) as it has a role in the upregulation of IL-1*β*, IL-6, IL-12, TNF-*α*, and MIP-1*α* production. This adipokine could also increase the immature DC migration and influence DCs' chemotactic activity and, finally, force the cells towards Th1 priming [45]. It should also be highlighted that leptin may have an impact on the DCs' survival by reducing apoptosis via modulation of NF-*κ*B (nuclear factor kappa-light-chain-enhancer of activated B cells), PI3K/Akt, Bcl-2, and Bcl-xL [46]. All the mentioned roles of leptin in DC functionality result in DCs' maturation and migration [4]. Simultaneously, DC's sensitivity to metabolic disturbances, including functional leptin deficiency, as seen in adiposity, may cause an impairment of dendritic cells and could be implicated in immunodeficiency associated with obesity [23, 47].

The involvement of leptin in adaptive immunity has also been confirmed. Leptin shifts the *T* helper balance towards a proinflammatory Th1 phenotype by stimulating proliferation of leptin receptor-expressing *T* cells, influencing proinflammatory cytokines and macrophages [23]. It promotes CD4+*T* cell polarization towards a proinflammatory Th1 phenotype, resulting in the secretion of IFN-*γ* and IL-2 [48]. Finally, there is a strong suggestion that leptin could also act as a negative signal and unfavorable regulator for the proliferation of Treg cells which normally suppress autoimmunity [23].

## 3. The Suggested Impact of Adipose Tissue on the Thyroid Autoimmunity and Thyroid Function

Although some findings indicate that adiposity may increase the risk of developing several autoimmune diseases, data confirming that obesity and thyroid autoimmunity are linked are scarce [17]. Nevertheless, it is still hypothesized that adipose tissue might play a role in autoimmunological processes related to the thyroid [17]. Undoubtedly, in obesity, chronic inflammation with a low-grade intensity is related to white adipose tissue activity [3]. Interestingly, animal studies revealed that adipokines/cytokines, especially TNF-*α*, may interact with an increased AITD rate in a mouse model [26, 49, 50].

It is known that adiposity triggers inflammatory processes [17] and, therefore, it is highly needed to explore all the pathways that may link adipose tissue and autoimmune reactions.

Firstly, alterations in adipokines secretion and activity may be responsible, at least partially, in generating or promoting a shift from Th2 to Th1 immune response that is related to autoimmune processes [51]. Among these adipokines taking part in this process, there are classical adipokines (e.g., leptin, adiponectin, and visfatin) and those that are regarded as cytokines [17].

Moreover, adipose tissue, mainly in visceral location, might be considered as the immune-regulating organ. The visceral adipose tissue comprises, in addition to typical adipocytes, of resident macrophages, endothelial cells, and *T* cells with biased *T* cell receptors [17, 52]. These immune-competent cells may control the immune response by exaggerating the release of proinflammatory cytokines. Specific *T* cells, Tregs, are also present within visceral adipose tissue and possess the ability to control autoimmune reactions [17, 23]. Interestingly, Treg cells could be influenced by leptin, and leptin could downregulate the proliferation of a Treg subpopulation involved in the control of autoimmunity and thyroid cell apoptosis [52–55].

**Figure 1:**
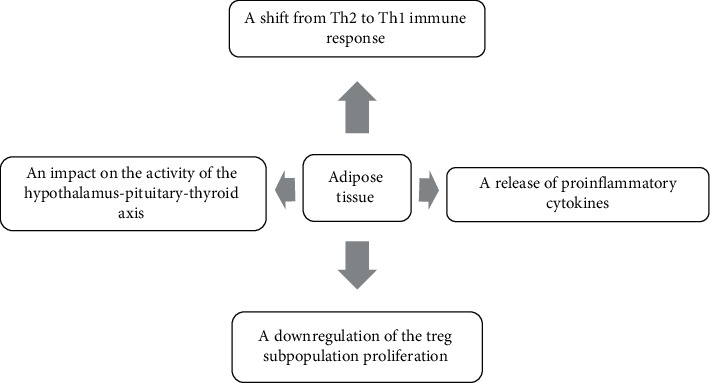
The potential impact of adipose tissue on the mechanism of thyroid autoimmunity and thyroid function.

Besides the role of adipokines and adipose tissue-derived cytokines in triggering inflammation, these molecules, as energy metabolism regulators, may have an impact on the activity of the hypothalamus-pituitary-thyroid (HPT) axis [17, 23, 56]. The potential impact of adipose tissue on the mechanism of thyroid autoimmunity and thyroid function is shown in Figure 1.

The best-known adipokine is leptin. Its activity acts as a signal to maintain suitable energy storage. Depending on the levels of leptin which are correlated with current nutritional status and energy reservoir, changes in the body homeostasis occur including those at the hypothalamic level. Low leptin concentrations result in conserving energy and increasing food intake. On the contrary, increased leptin levels related to adiposity should let to reduce food consumption and enhance energy utilization by modulation of the neuroendocrine pathways [26, 57]. Then, leptin links nutritional status with neuroendocrine and immune function [58].

The main action site of leptin is the arcuate nucleus (ARC) located in the mediobasal part of the hypothalamus [59]. It should be highlighted that arcuate neurons project to thyroid-releasing hormone (TRH) neurons [60]. Besides, leptin receptors (Lep-R) have also been found in the pituitary gland and on the TRH-secreting neurons of the paraventricular nucleus (PVN) [59]. Furthermore, findings confirm the hypothesis that leptin signaling is necessary for the maintenance of TRH expression in the hypothalamic PVN and, consequently, for normal production of TSH (thyroid-stimulating hormone) and subsequently thyroid hormones [23, 61]. Moreover, in lean healthy subjects, the circadian rhythms of TSH and leptin may overlap [62]. It has been supposed that leptin may regulate, at least in euthyroid individuals, not only basal TSH secretion but also its pulsatility and circadian clock pattern [23, 60, 62]. Therefore, improper daily leptin secretion caused by under- or overnutrition may change the activity of the hypothalamus-pituitary-thyroid axis by influencing the hypothalamic network.

Interestingly, decreased leptin levels mediate downregulation of the hypothalamus-pituitary-thyroid axis by disrupting TRH release in the hypothalamus [63]. Experimental data, obtained from the murine model of fasting, indicated that leptin administration abolished the reduction of TRH expression in the hypothalamus and, additionally, increased the hypothalamic deiodinase type 2 (D2) expression [64, 65]. The effect of leptin on the pituitary TSH expression was less prominent. Furthermore, leptin action on the TRH neurons in the PVN can be direct (as an effect on TRH neurons expressing Lep-R) or indirect [17]. The indirect effect of leptin may involve alpha-MSH production in POMC (pro-opiomelanocortin) neurons of the ARC-targeting TRH neurons [66]. Indeed, under fasting conditions, TRH mRNA is reduced, and this decrease is mediated by the suppression of alpha-MSH/CART (*α* melanocyte-stimulating hormone/cocaine and amphetamine-regulated transcript) simultaneously with an increase in NPY/AGRP (neuropeptide Y/agouti-related protein) gene expression in the ARC neurons [67]. As a result of fasting and changes in the neuroendocrine circuit, a decline in thyroid hormone levels is observed and the phenomenon is presumably related to enhanced TRH gene sensitivity to negative feedback inhibition by thyroid hormone [67].

Taken together, the abovementioned data support the hypothesis that adipose tissue, by adipose-derived peptides and cytokines, influences thyroid function with particular reference to the autoimmunological processes.

## 4. An Influence of TSH and Thyroid Hormones on the Activity of Adipose Tissue

According to the clinical and experimental studies, thyroid hormones might influence the activity of adipose tissue and, consequently, there could be a bilateral interaction between thyroid and adiposity [17]. Firstly, it is widely accepted that the actual nutritional status, body weight, and energy expenditure may influence thyroid hormone secretion [68]. Undoubtedly, chronic starvation is associated with changes in hormone concentration. Extended caloric deprivation as seen in anorexia nervosa results in a decrease in total and free T4 (thyroxine) and T3 (triiodothyronine) and an increase in rT3 that resemble the euthyroid sick syndrome [69]. Furthermore, the TSH response to TRH is diminished and, in some cases, thyroid-binding protein levels are decreased [69]. Interestingly, hypocaloric diets as a treatment of obesity cause changes in thyroid function mirroring thyroid activity in the course of the euthyroid sick syndrome [69].

Selected pathological conditions with deficits or overproduction of thyroid hormones result in an impaired amount of fat tissue and metabolic alterations. Hyperthyroidism is correlated with a hypermetabolic state. Due to an excess of thyroid hormones, several metabolic disturbances are seen including an increase in resting energy expenditure (REE), reduced cholesterol levels, increased lipolysis, and gluconeogenesis resulting in weight loss [68, 70]. Triiodothyronine has an impact on white adipose tissue, especially on its lipolytic activity, and this effect is mediated by the cAMP-dependent mechanism and is synergized by the adrenergic system [17, 71]. Even though thyroid hormones stimulate both lipogenesis and lipolysis, in hyperthyroidism, the final effect of thyroid hormone excess is a reduction of adipose tissue amount [68, 72].

On the other hand, a decrease in thyroid hormones secretion seen in hypothyroidism leads to hypometabolism in which metabolic changes are just opposite to those observed in hyperthyroidism (reduced resting energy expenditure, weight gain, increased cholesterol levels, reduced lipolysis, and reduced gluconeogenesis) [68].

Undoubtedly, there is a close connection between thyroid hormones and energy balance. Thyroid hormones not only influence the energy expenditure but also have an impact on appetite regulation through the central nervous system, mainly the hypothalamus, as well as on the fat tissue, skeletal muscles, liver, and pancreas. In detail, specific thyroid hormone activity is responsible for key metabolic pathways that comprise the molecular mechanisms, lipid regulation, crosstalk with nuclear receptors, the role of corepressors in metabolic regulation, thyroid hormone adrenergic interactions, facultative thermogenesis, and the metabolic influences on the central regulation of thyroid hormones [68].

Thyroid hormones are necessary for the full thermogenic response of BAT (brown adipose tissue), and normal systemic thyroid function is essential for cold-induced adaptive thermogenesis [73]. Thyroid activity may determine thermogenesis in different ways. The hypothalamic-pituitary-thyroid axis regulates genes that influence thermogenesis in BAT [17, 74]. Moreover, thyroid hormones might bind to the different isoforms of the thyroid hormone receptors that are expressed on both brown and white adipose tissue cells, including a1, a2, and b1 [17]. Furthermore, thyroid hormones play a role in an adaptive (named also facultative) no shivering thermogenesis [17]. Indeed, during cold exposure, the thyroid hormone-activating enzyme type 2 deiodinase increases the generation of T3 in brown adipose tissue. The thermogenic effect is mediated by the uncoupling protein 1 (UCP1) and possibly by the UCP3 [17]. The type 2 iodothyronine deiodinase plays a critical role in modulating the amount of the active T3 in BAT, thereby modulating the responses to impulses from the sympathetic nervous system [75]. Possibly, an additional mechanism generating an increased turnover of calcium in the sarcoplasmic reticulum could also be involved [75]. Besides, thyroid hormones also affect the hypothalamus where specific thyroid receptors are found, and by binding to those receptors, thyroid hormones modulate the sympathetic nervous output to BAT. Moreover, triiodothyronine at the hypothalamic level decreases the activity of hypothalamic AMP-activated protein kinase and increases sympathetic nervous system activity. Additionally, T3 upregulates thermogenic markers in brown adipose tissue [76]. Therefore, the thermogenic response of BAT to thyroid hormone input results from the synergistic interactions of the hormones with the sympathetic nervous system [73]. On the other hand, exposition to low temperatures markedly activates the HPT axis and increases TRH synthesis, TSH release, and serum thyroid hormone concentrations. All these mechanisms coordinate an increase in thermogenesis and cold adaption [77].

Interestingly, there is increasing evidence for the role of thyroid hormones in the process of white adipose tissue browning. Beige adipocytes are multilocular with moderate mitochondrial content and inducible expression of UCP1. They form within the white adipocyte depots in response to several conditions including chronic cold and exercise. Although some reports indicated that beige adipocytes, when fully stimulated, can functionally mimic the metabolic actions of classical brown adipocytes, the physiological role of this kind of adipocytes has not been fully elucidated [78–80].

It is worth to notice that preliminary human studies have confirmed the interaction between thyroid status and brown adipose tissue activity. Although it was observed that in euthyroid volunteers BAT activation is not related to peripheral thyroid hormone concentrations, it was indicated that BAT activity is influenced by higher TSH levels [81, 82]. The study on an adolescent with severe primary hypothyroidism revealed that BAT was abundantly present in the supraclavicular fossa and the supraclavicular temperature was higher than that in the suprasternal area. After treatment with l-thyroxine and obtaining euthyroid status, the BAT amount decreased and the temperature was homogenous [83]. On the contrary, it has been observed in another study that girls with autoimmune hypothyroidism presented an attenuated thermogenic response to cold stimulation when compared with healthy controls. However, those individuals with suboptimal biochemical control (with higher TSH) showed increased BAT activation. The authors suggested that the underlying thyroid disease may have a negative effect on BAT response, but high levels of TSH can mitigate, and even stimulate, BAT activity [84]. Not surprisingly, it has also been found that BAT activity is increased in the course of hyperthyroidism. In detail, TSH correlated inversely with BAT glucose metabolism but hyperthyroidism did not affect BAT perfusion [85].

Finally, TSH itself could also stimulate thermogenesis. Briefly, TSH receptors are present on the adipocytes and, consequently, some studies suggested that TSH, by binding to its receptor on brown adipocytes, may stimulate thermogenesis [74, 86, 87]. This mechanism could be involved in maintaining thermal status during hypothyroidism [17].

The presence of functional TSH receptors on the adipocyte surface may have another role compared to thermoregulation only. Data from the experimental study showed that the in vivo acute administration of recombinant human TSH at supraphysiological doses induced the release of small but significant amounts of leptin. A rise in leptin levels was proportional to the adipose mass [88]. Nevertheless, the exact role of TSH receptor expression on the surface of white adipocytes needs further investigation [17].

To sum up, a positive feedback mechanism between leptin and TSH indicates that TSH may serve as a modulator of adipocyte activity. Moreover, data from the literature may implicate that triiodothyronine could also influence adipose tissue metabolism as the thyroid hormone-activating enzyme type 1 deiodinase (D1) expression was found to be elevated in omental and subcutaneous fat and has been positively associated with leptin in obese individuals [89]. T3 produced via D1 in response to leptin plays a modulatory role in adipose tissue metabolism [23].

## 5. Genetic Aspects of AITD and Adiposity

Undoubtedly, genetic factors contribute both to AITD and obesity susceptibility. However, these diseases arise from the interactions between an at-risk genetic profile and environmental risk factors.

Nowadays, molecular methods have been used to identify the role of specific genes and their genetic variants in predisposition to multifactorial diseases including autoimmune thyroid disease and obesity. Molecular approaches include analysis of sequence variants analyzing single nucleotide polymorphisms (SNPs) in candidate genes and GWA (genome-wide association) studies, which associate DNA traits to particular pathological conditions [90, 91].

Data from studies on monozygotic twins with AITD provided robust evidence for the contribution of genetic factors to AITD susceptibility [91]. Further studies with the use of SNPs and GWA widen the knowledge about genes involved in the AITD pathogenesis. Amongst these genes, two major groups could be distinguished: thyroid-specific (Tg, TSHR) and immune-modulating (FOXP3, CD25, CD40, CTLA-4, and HLA), with HLA-DR3 carrying the highest risk [92]. FOXP3 and CD25 are critical factors of peripheral tolerance while CD40, CTLA-4, and HLA genes play a pivotal role in T lymphocyte activation and antigen presentation [92]. In GWAS of autoimmune thyroid diseases, many more susceptibility loci for AITD were detected as well. These genes are involved in *T*-cell signaling (PTPN22), thyroid morphogenesis (FOXE1), cytokine signaling (SH2B3), cytoskeletal rearrangements and transcriptional alterations (VAV3), regulation of actin filament dynamics (CAPZB), hydrolysis of the second messenger cAMP (PDE8B), apoptosis of hematopoietic cells (TRIB2), and cell-cell adhesion and cell motility (LPP) [93]. Through GWAS of various thyroid-related phenotypes, some associations have been commonly identified in people of different geographical regions, while many other susceptibility loci have been found only in specific populations [93].

Similar to AITD, obesity is a complex disease, associated with different susceptibility loci and environmental risk factors including physical inactivity, excessive caloric intake, the intrauterine environment, medications, socioeconomic status, and possibly novel factors such as insufficient sleep, endocrine disruptors, and the gastrointestinal microbiome [94]. Additionally, rare monogenic forms of human obesity have been described, mainly point-mutations in the leptin, POMC, or melanocortin 4 receptor (M4CR) genes [90].

Several individual single nucleotide polymorphisms (SNPs) in human genes have been linked to obesity risk. The importance of some genes encoding the obesity-related products has been reported [90]. Furthermore, studies revealed BMI- (body mass index-) and WHR- (waist-hip ratio-) associated loci that are related to higher insulinogenic indices [90].

In addition to SNPs, structural traits of DNA have been also highlighted by GWAS. According to the review from 2018, major GWAS results for adiposity traits are grouped into the following seven categories:

1. BMI-related (includes GWAS for BMI, weight, overweight or obese status in adulthood, childhood BMI, childhood obesity, and BMI change over time; 141 loci)
2. Body fat (includes GWAS for body fat percentage and body fat mass; 15 loci)
3. Birthweight (eight loci)
4. The waist-to-hip ratio or waist circumference adjusted for BMI (97 loci)
5. Visceral adiposity (includes GWAS for visceral fat and visceral-to-subcutaneous adipose tissue ratio; two loci)
6. The waist-to-hip ratio or waist circumference (includes GWAS for waist-to-hip ratio and waist circumference not adjusted for BMI; 26 loci)
7. Extreme obesity (includes GWAS for extreme childhood and extreme adult obesity; 23 loci) [94]

The role of epigenetic regulators (as transcriptional coregulators, miRNAs, and long noncoding RNAs) on the homeostasis of adipose tissue cannot be omitted. The impact of epigenetic regulators is critical since surgical, pharmacological, or dietary interventions could be insufﬁcient in overcoming the epigenetic reprogramming of key metabolic genes [90].

From the clinical point of view, four basic types of obesity could be distinguished according to body mass and metabolic profile: metabolically healthy nonobese (MHNO), metabolically unhealthy nonobese (MUNO), metabolically healthy obese (MHO), and metabolically unhealthy obese (MUO). A subset of patients with obesity who display a metabolically healthy phenotype is characterized by insulin sensitivity, as well as normal blood pressure and normal lipid and inflammatory profiles. It could be suspected that genetic predisposition and lifestyle factors can influence such phenotypes [95]. Findings from experimental studies in animal models have shown that adipose tissue expandability, fat distribution, adipogenesis, adipose tissue vascularization, inflammation and fibrosis, and mitochondrial function are the main mechanisms that uncouple obesity from its metabolic comorbidities [96]. Up to date, no GWAS results are confirming genetic variants associated with obesity phenotypes, especially MHO or MUNO. However, it should be highlighted that there are known genetic variants associated with increased adiposity and, simultaneously, with a favorable cardiometabolic profile (e.g., lower glucose levels, lower triglyceride and higher HDL-cholesterol levels, lower blood pressure, and/or lower risk of T2D and coronary artery disease), the features seen in the MHO phenotype [96]. On the contrary, alternate allele is associated with decreased adiposity and an unfavorable cardiometabolic profile similar to that observed in the MUNO phenotype [96]. Moreover, genome-wide association studies that aimed to identify variants for body fat % (BF%) indicated that, for some of the variants, the BF%-increasing allele has protective effects on cardiometabolic outcomes [96]. Furthermore, insulin resistance-associated loci, individually or aggregated, are linked to higher cardiometabolic risk, despite being associated with a decreased amount of adipose tissue [96]. Besides, genetic variants of lipid regulatory genes have been associated with the metabolically unhealthy phenotype of obese individuals [95]. Although GWASs have identified numerous novel adiposity loci, the biology that these loci represent should be elucidated extensively [97].

Unfortunately, to date, there are no available studies concerning genetic variants that could be associated with thyroid autoimmunity and, at the same time, adiposity.

## 6. Clinical Correlations of Thyroid Function, AITD, and Adipose Tissue

At least in theory, based on the reported role of adipose tissue in the development of autoimmunity, there should be a link between obesity and the presence of Hashimoto's disease. However, up to date, an unequivocal association between adiposity and autoimmune thyroid disease has not been established [8, 17]. Interestingly, there is also neither clear thyroid hormones pattern nor typical layout of conventional markers of thyroid autoimmunity, such as thyroid peroxidase antibodies and/or ultrasound thyroid hypoechogenicity in obese individuals [17].

Therefore, several questions could be raised. The first issue concerns if there is any correlation between TSH, FT3 (free triiodothyronine), and FT4 (free thyroxine) levels and adiposity measurements. The second important question is whether there is a greater prevalence of HT-related autoantibodies among obese individuals and, finally, whether there are changes in ultrasound thyroid structure in subjects suffering from obesity.

Although thyroid function was extensively investigated in many clinical studies with the participation of obese individuals with or without the presence of the thyroid antibodies, the obtained data provided discrepant results.

Studies concerning the relationship between serum TSH concentrations and BMI as a marker of adiposity mostly indicated a positive correlation between those two parameters [8]. A recently published systematic review and meta-analysis of 22 studies by Song et al. showed that obesity was significantly associated with an increased risk of overt and subclinical hypothyroidism. Moreover, adiposity was associated with Hashimoto's thyroiditis, but not with Graves' disease [98]. Similar results were presented in another review in which 29 studies were analyzed [99]. The majority of the presented results, 18 out of 29, confirmed the positive association between TSH and BMI [99]. Furthermore, findings from the National Health and Nutrition Examination Survey 2007–2008, on a representative sample of the adult U.S. population, also showed a significant correlation between TSH and two indicators of adiposity: BMI and waist circumference measurements [100]. Another meta-analysis with the evaluation of TSH levels in obese individuals revealed the trend to high, but still within the normal range, concentrations of TSH being associated with enhanced BMI [101]. Very recently, a large population-based study from Taiwan also confirmed that elevated TSH (but within the normal range) was related to central obesity as TSH levels were dose-dependently associated with increased body mass index, body fat percentage, and waist circumferences [102]. However, other authors of the cross-sectional study that included more than 5000 euthyroid individuals failed to confirm any association between TSH and BMI [103].

Contrary to the data presented above, recently published results of a large population study on the association of TSH levels and visceral adipose tissue (VAT) showed no correlation [104]. In detail, the study conducted by Witte and colleagues involved a sample of near 2000 participants in whom visceral adipose tissue volume was measured using magnetic resonance imaging. Although the authors failed to find any association between TSH and visceral adipose tissue, they indicated that VAT was strongly related to leptin with a greater effect in women than in men and, not surprisingly, leptin showed a strong correlation with TSH [104]. Lack of direct association between TSH and adipose tissues in different locations (including whole body mass, visceral and subcutaneous area, and visceral fat thickness) was also confirmed in the studies in which other techniques compared to MRI were used, e.g., computer tomography or indirect measurement of preperitoneal fat with ultrasound [105–107].

Intensive research on peripheral thyroid hormone concentrations in obese individuals also resulted in inconsistent findings. Data regarding serum levels of FT3 presented increased, unchanged, or decreased FT3 concentrations in the course of obesity [17]. On the other hand, findings from the National Health and Nutrition Examination Survey indicated a positive correlation between FT3 and both BMI and waist circumferences [100]. Contrary to FT3, FT4 levels in another study showed a trend towards low or normal values [17]. Interestingly, no association between FT4 and BMI or waist circumference was observed in the National Health and Nutrition Examination Survey [100]. The explanation of the tendency to lower levels of FT4 in obese individuals may include the inhibiting impact of leptin on the thyroxine secretion [23].

The issue often rose by patients as well as by scientists is the problem of weight gain in the course of hypothyroidism. In one of the cohort studies, more than 50% of hypothyroid patients complained about weight excess [108]. However, the part of the Rotterdam study that involved elderly women suffering from subclinical hypothyroidism indicated no difference in BMI of hypothyroid subjects in comparison with euthyroid controls [109]. On the other hand, it has been suggested that changes in body weight in hypothyroid patients are associated not only with the volume of fat mass measured with bioimpedance [110–112], decreased REE [113], and reduced physical activity [114, 115] but also depend on impaired ability to excrete free water [116] and increased amounts of glycosaminoglycans [117] that are involved in the greater water-binding capacity.

Another problem that needs to be discussed is the relationship between obesity and the occurrence of chronic autoimmune thyroiditis. Herein, data from the literature also showed conflicting results. Despite some findings that severely obese individuals have high but still normal TSH levels, it has been suggested that these results are not related to the autoimmune process as the low prevalence of HT-related autoantibodies was found [118]. Furthermore, the group of Amouzegar observed no significant differences in TPOAb (thyroid peroxidase antibody) levels between the various obesity phenotypes [119]. Also, the newest study of Amouzegar et al., in which different metabolic types of abdominal obesity were evaluated, revealed that none of the phenotypes or higher waist circumference was associated with increased risk of developing TPOAb positivity [2].

On the contrary, other authors suggested that obese people could more often develop autoimmune thyroiditis [8]. Song and colleagues indicated in the meta-analysis that obesity was correlated with positive thyroid peroxidase antibody but not with positive thyroglobulin antibody (TgAb) [98]. Besides, another cohort study showed that the prevalence of positive TPOAb and TgAb had a statistically significant rising trend with the increase in overweight/obesity grade measured with BMI and waist circumference both in males and females. However, abdominal obesity in males was significantly associated with positive TgAb risk after adjusting for the confounding factors [120].

Other cohort studies also showed a greater prevalence of hypothyroidism with HT-related autoantibodies among obese individuals. The study by the group of Ong suggested that overweight in childhood led to a slightly increased risk of HT at the age of 60–64 years, particularly in women [121]. Besides, the results of the cross-sectional epidemiological study that included 1317 healthy subjects aged 2–16 years also confirmed that the prevalence of thyroid autoimmunity was lower in young individuals with normal weight than in their overweight and obese counterparts [122].

In addition, the outcomes of the cohort study on the impact of obesity on the risk of hypothyroidism and thyroid autoimmunity among Chinese adults indicated that there were sex differences in the associations of obesity with hypothyroidism and thyroid autoimmunity. In detail, a significant association of obesity with hypothyroidism and subclinical hypothyroidism was seen in females but no association between obesity and hypothyroidism was observed in male participants. Furthermore, BMI was significantly and positively correlated with TPOAb in men but not in women. Linear regression analysis suggested a negative correlation of BMI with TgAb in women. The authors explained these results by the sex differences in body fat distribution, the influence of sex hormones, the role of obesity in changes of sex hormones profiles, and, finally, known differences in the adipokine levels between men and women. [123]. On the other hand, the novel findings from a population-based cross-sectional study on 12531 Chinese individuals conducted by Guo and coworkers indicated that overweight and obesity were associated with hyperthyrotropinaemia only in the presence of thyroid autoimmunity [124]. Furthermore, the group of Marzullo showed a greater prevalence of hypothyroidism and the presence of HT-related autoantibodies (TPOAb) among obese patients, and these clinical findings were correlated with increased leptin levels. Of note, leptin levels were associated with AITD independently of anthropometric variables [125].

When considering the current data related to the activity of leptin in autoimmune processes and novel findings of higher leptin levels in patients with Hashimoto's thyroiditis, which positively correlated with the percentage of Th17 cells [126], it could be stated that adipose tissue-derived leptin is an important player in autoimmune thyroiditis. Indeed, findings from the study of Drobniak and coworkers indicated the significant differences in leptin levels between women with AITD and their counterparts without AITD. Interestingly, BMI was comparable between the groups [127]. On the other hand, Delitala et al. showed in their cohort of the Sardinian population that neither leptin nor BMI was associated with the presence of any circulating thyroid antibody [103].

Furthermore, longitudinal population-based studies broaden our knowledge in the field of the relationship between thyroid and adiposity. The recently published cohort study by Abdi and coworkers, based on the findings of the Tehran thyroid study, showed that in normal-weight individuals, after an average follow-up of 9.8 years, the risk of having an abnormal BMI (indicating being overweight or obese) increased with a decrease in serum FT4 concentrations. However, no association was observed with the change in serum TSH levels or the change in TPOAb status [128].

On the contrary, findings of other longitudinal studies revealed conflicting results as TSH is positively related to enhanced BMI in the majority of the reports [129–135]. Moreover, FT4 did not show any association with weight changes in the study by Knudsen, but an inverse association between FT4 change and weight change was found in the DanThyr study [135]. Data from another part of the Tehran Thyroid Study strongly suggested that serum FT4 concentrations within the reference range were associated with the development of the MHNW and MHO (metabolically healthy) obesity phenotypes [119]. Interestingly, the group of Soriguer demonstrated that, among nonobese individuals, the risk of becoming obese at the 6-year follow-up increased significantly for those in whom baseline FT3 and FT4 were in the fourth quartile [131].

Notably, thyroid volume, structure, and echogenicity, assessed in the ultrasound examination, may change in the course of obesity independently of the presence of autoimmune processes. The gland volume in obese patients measured by ultrasound was greater than in nonobese individuals and this difference was related to the amount of lean body mass but not to body mass [136]. Interestingly, after weight loss, the thyroid volume was reduced [137]. Besides, a significant improvement of the thyroid echogenicity was found in previously morbidly obese and euthyroid adults as a result of weight loss due to bariatric therapy [138].

When considering the thyroid echogenicity in children and adults with obesity, the relation of this feature with thyroid autoimmunity should be taken into account. However, only 20% of morbidly obese individuals, in whom hypoechogenic thyroid was observed, presented thyroid autoimmune antibodies in opposite to the nonobese controls with similar ultrasound patterns in whom a greater prevalence of thyroid antibodies was found [139]. Moreover, in obese children, alterations of thyroid structure with an ultrasound pattern suggestive of Hashimoto's thyroiditis were observed but these findings could not be completely explained by the presence of an autoimmune involvement [140].

## 7. Conclusions

**Figure 2:**
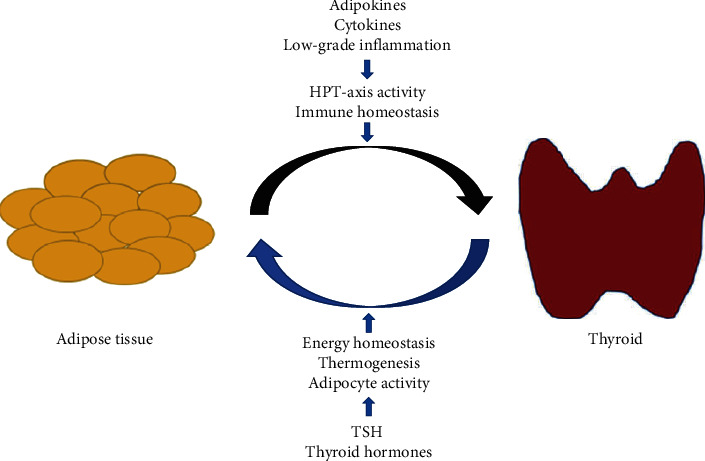
The bilateral relationship between autoimmune thyroid disease/thyroid function and adiposity.

Data in the literature suggests that there could be a reciprocal relationship between the thyroid and adipocytes (Figure 2).

Findings from both experimental and clinical studies indicate that adiposity may influence the onset and course of autoimmune disease. The discrepancies observed between the studies, especially when considering the relationship between TSH and obesity, result from confounding factors such as age, sex, smoking, iodine intake, and distribution of body fat (either subcutaneous or visceral), making the results of the studies difficult to compare. Therefore, further intensive research with careful consideration of the abovementioned confounders is needed to explain all the aspects of the relationship of adiposity and autoimmune thyroid disease. It may be necessary to consider obesity-related factors other than those that have so far been examined. Metabolic markers or adipose tissue features related to inflammation should presumably be considered. Furthermore, it could be hypothesized that examination of pathophysiologic mechanisms involved in forming different obesity phenotypes would allow for understanding of underlying processes involved in thyroid autoimmunity-obesity and thyroid function-obesity interactions.

## Data Availability

No data were used to support this study.
